# Association of Incarceration With Mortality by Race From a National Longitudinal Cohort Study

**DOI:** 10.1001/jamanetworkopen.2021.33083

**Published:** 2021-12-23

**Authors:** Benjamin J. Bovell-Ammon, Ziming Xuan, Michael K. Paasche-Orlow, Marc R. LaRochelle

**Affiliations:** 1 Section of General Internal Medicine, Boston Medical Center, Boston, Massachusetts; 2 The Miriam Hospital, Lifespan, Providence, Rhode Island; 3 Department of Community Health Sciences, Boston University School of Public Health, Boston, Massachusetts; 4 Boston University School of Medicine, Boston, Massachusetts

## Abstract

**Importance:**

The association between incarceration and long-term mortality risk is unknown and may contribute to racial disparities in overall life expectancy.

**Objective:**

To determine whether incarceration in the US is associated with an increase in mortality risk and whether this association is different for Black compared with non-Black populations.

**Design, Setting, and Participants:**

This generational retrospective cohort study used data from the National Longitudinal Survey of Youth 1979, a nationally representative cohort of noninstitutionalized youths aged 15 to 22 years, from January 1 to December 31, 1979, with follow-up through December 31, 2018. A total of 7974 non-Hispanic Black and non-Hispanic non-Black participants were included. Statistical analysis was performed from October 26, 2019, to August 31, 2021.

**Exposures:**

Time-varying exposure of having experienced incarceration during follow-up.

**Main Outcomes and Measures:**

The main outcome was time to death. Cox proportional hazards regression models were used to estimate hazard ratios (HRs) and 95% CIs, adjusted for baseline sociodemographic, economic, and behavioral risk factors. Models were evaluated for the full cohort and stratified by race.

**Results:**

Of the 7974 individuals included in our sample, 4023 (50.5%) were male, and 2992 (37.5%) identified as Black (median age, 18 [IQR, 17-20] years). During a median follow-up of 35 years (IQR, 33-37 years), 478 participants were incarcerated and 818 died. Unadjusted exposure to at least 1 incarceration between 22 and 50 years of age was 11.5% (95% CI, 10.4%-12.7%) for Black participants compared with 2.5% (95% CI, 2.1%-2.9%) for non-Black participants. In the multivariable Cox proportional hazards model with the full cohort, time-varying exposure to incarceration was associated with an increased mortality rate (adjusted HR [aHR], 1.35; 95% CI, 0.97-1.88), a result that was not statistically significant. In the models stratified by race, incarceration was significantly associated with increased mortality among Black participants (aHR, 1.65; 95% CI, 1.18-2.31) but not among non-Black participants (aHR, 1.17; 95% CI, 0.68-2.03).

**Conclusions and Relevance:**

In this cohort study with 4 decades of follow-up, incarceration was associated with a higher mortality rate among Black participants but not among non-Black participants. These findings suggest that incarceration, which was prevalent and unevenly distributed, may have contributed to the lower life expectancy of the non-Hispanic Black population in the US.

## Key Points

**Questions:**

Is exposure to incarceration associated with a long-term increase in mortality rate, and does this association differ by race?

**Findings:**

In this cohort study of 7974 individuals who were followed up from 1979 to 2018, incarceration was associated with a 65% higher mortality rate among Black participants. Among non-Black participants, incarceration was not associated with mortality.

**Meaning:**

These findings suggest that racial disparities in the association of incarceration with mortality—as well as in rates of exposure to incarceration—may partially explain the lower life expectancy of the non-Hispanic Black population in the US.

## Teaser

This cohort study assesses whether incarceration during adulthood is associated with a higher mortality rate and whether this association differs by race.

## Introduction

During the era of mass incarceration of the last 4 decades, the incarcerated population in the US has quadrupled in size,^1^ and the US incarcerates a greater number and proportion of its citizens than any other country in the world.^2,3^ As a manifestation of structural racism—which Bailey et al define as “the totality of ways in which societies foster racial discrimination, via mutually reinforcing inequitable systems…that in turn reinforce discriminatory beliefs, values, and distribution of resources”^4^^(p1455)^—this growth in carceral practices has fallen disproportionately on specific minoritized racial groups, particularly Black men.^5,6^ Serving time in prison during young adulthood is as common for Black men as college graduation is for White men.^7^

Life expectancy is worse in the US than in most other high-income countries and has some of the widest disparities across race and class.^8,9^ The experience of incarceration, which is linked to numerous ill health effects, may contribute to racial health disparities and to the relatively poor health of the population as a whole.^10,11,12^ A variety of mechanisms may explain this connection to health, from the stressful disruption of detention and release, to the enduring effects of stigma, decreased earning potential, frayed social bonds, and other challenges that persist long after a person returns home.^13,14,15^

However, the true effect of incarceration on life expectancy in the US has been difficult to measure. Studies have documented the increased mortality rates of individuals recently released from prison or jail, particularly due to drug overdose,^16,17^ but few have examined long-term mortality. Because many of the risk factors for incarceration (eg, poverty, low educational level, experiences of racism) are also social determinants of poor health, it is difficult to disentangle the effect of incarceration from the health effects of these other factors. The longitudinal data that would be necessary to elucidate these associations are limited, because incarceration and involvement in the criminal-legal system are largely ignored in most national health studies.^18^ In this study, we used one of the longest-running national cohort studies with data on incarceration and a wide variety of other social determinants of health. We sought to determine, first, whether the experience of incarceration during the life course is associated with a higher mortality rate when adjusting for baseline markers of social and economic status, and second, whether the association of incarceration with mortality differs by race.

## Methods

### Study Design, Data Source, and Cohort Selection

We conducted a retrospective cohort study using data from the National Longitudinal Survey of Youth 1979 (NLSY79). Administered by the US Bureau of Labor Statistics, NLSY79 recruited a nationally representative cohort of noninstitutionalized youth from January 1 to December 31, 1979, who were 15 to 22 years of age, and who have been followed up ever since.^19,20^ Data were collected via in-person or telephone surveys administered annually from 1979 to 1994 and every 2 years after that. We used data from all 28 survey rounds conducted through December 31, 2018. The Boston University Medical Campus institutional review board determined that this study was not human participant research and did not require informed consent. Reporting in this study followed all applicable Strengthening the Reporting of Observational Studies in Epidemiology (STROBE) guidelines.

In this study, we included participants if they were in a subsample that was retained in NLSY79 throughout the study period—namely, the cross-sectional civilian sample, the supplemental oversample of non-Hispanic Black participants, and the retained portion of the military sample. We excluded 2 supplemental subsamples that NLSY79 dropped in the first decade owing to funding changes (see eMethods 1 and eFigure 1 in the Supplement for details).

Using the 3 categories defined by NLSY79’s main race and ethnicity variable—Hispanic (of any race), non-Hispanic Black, and non-Hispanic non-Black—we limited our analysis to only the non-Hispanic Black (hereafter, Black) and non-Hispanic non-Black (hereafter, non-Black) groups for clarity of comparison. This race and ethnicity variable relied partly on interviewer-perceived race in addition to participant self-identification and did not allow for reliable differentiation between non-Hispanic White and other non-Hispanic non-Black groups (eg, Asian, Native American) (see eMethods 2 and eTables 1 and 2 in the Supplement for details on our approach to the available race and ethnicity data).^21,22^

We indexed all longitudinal survey data by participant age in years (rather than calendar year) and started follow-up at 22 years of age, the youngest age with baseline data available for the entire cohort (eFigure 2 in the Supplement). We considered the baseline period to include the surveys completed by a participant at or before 22 years of age. We excluded participants who died or were lost to follow-up before 22 years of age. Participants remained in our study cohort until death or censoring (loss to follow-up or the end of the study period).

### Key Variables

The outcome was death, identified as the reason for noninterview in survey rounds after a participant’s death. We considered participants to be alive up until the year they were recorded as deceased in a survey response.

For the exposure, we used 3 sets of survey variables to identify incarcerations: in 1980, survey participants were asked if they had ever been incarcerated and, if so, the date of their last release; in subsequent surveys, we identified incarceration if a participant’s residence was recorded as jail/prison or if they were not surveyed and the reason recorded was incarceration. We note that incarceration as a possible reason for noninterview was first introduced with the 2004 survey. Our primary exposure was time-varying incarceration after 22 years of age, the start of follow-up for these analyses. This design established a uniform time zero at which to identify baseline characteristics that are potential confounders and from which to follow up for the exposure and outcome of interest. We operationalized incarceration as a yearly time-dependent dichotomous exposure variable: participants were considered unexposed to follow-up incarceration until they experienced an incarceration in this period and were considered exposed thereafter. We characterized any incarceration before 22 years of age in a separate baseline variable, prior incarceration.

We identified important baseline characteristics that may be potential confounders. We included participants’ sex. We defined baseline disability as reporting a health condition that prevented one from working or limited the amount or type of work one could do. We identified whether participants received a high school diploma or equivalent by 22 years of age. We dichotomized parental high school completion as both parents completed high school vs at least 1 parent did not complete high school or had missing data. As a marker of poverty, we identified whether participants reported income from public assistance or welfare programs during the baseline period. We scaled total annual family income as a percentage of the year-specific federal poverty level and used the mean of all available values during the baseline period. From the 1980 survey, we assessed whether participants reported any illicit drug use and any engagement in illegal activity in the past 12 months.

### Statistical Analysis

Statistical analysis was performed from October 26, 2019, to August 31, 2021. We analyzed both time to incarceration and time to death. We used cumulative incidence function estimates, which accounted for the competing risk of death^23^ to generate unadjusted cumulative exposure to incarceration curves and tested for between-group difference by race using the Gray nonparametric test.^24^ We modeled the cumulative incidence function of incarceration exposure using a proportional subdistribution hazards regression model accounting for the competing risk of death^25^ and adjusted for the following baseline characteristics: prior incarceration, race, sex, disability, income, receipt of public assistance, participant high school completion, parental high school completion, drug use, and illegal activity. For the time-to-death analysis, we used an extended Kaplan-Meier estimator^26^ allowing for time-varying incarceration exposure to generate unadjusted cumulative incidence of death curves. Extended Kaplan-Meier curves represent hypothetical cohorts whose exposure statuses remain constant throughout follow-up (so they are not to be interpreted as the percentage of a real cohort with an event over time).^27,28^ We modeled time to death using a standard Cox proportional hazards regression model with follow-up incarceration as a time-dependent exposure variable and the other covariates included in the time-to-incarceration model. To evaluate whether race modifies the effect of incarceration on mortality, we analyzed race-stratified versions of the model. Due to the NLSY79’s cluster-sampling design, we included sample weights and adjusted our time-to-death regression models to account for potential similarities among participants within each geographic cluster (ie, primary sampling unit). For any significant association between incarceration and mortality, we calculated E-values to assess the minimum strength of association (expressed on a hazard ratio [HR] scale) that an unmeasured confounder would need to have with both incarceration and mortality, controlling for all measured covariates, to explain away the observed association.^29^

We used multiple imputation with 10 imputations to account for missing data among baseline covariates. We further examined the impact of potentially informative missingness by performing a complete case analysis (ie, excluding participants with any missing covariate data from the sample). To assess the impact of excluding 2 of the NLSY79’s original subsamples (eMethods 1 in the Supplement), we performed a sensitivity analysis by including all non-Hispanic subsamples, censoring participants if or when their subsample was dropped early by the NLSY79. To examine the impact of specifying 2 separate incarceration variables, we conducted a sensitivity analysis by creating a unified time-dependent incarceration variable that considered participants with baseline prior incarceration to be exposed at the start of follow-up. We used SAS, version 9.4 (SAS Institute Inc) for all analyses. Two-sided *P* < .05 indicated statistical significance.

## Results

### Baseline Characteristics

Of 12 686 participants in the NLSY79 cohort, we identified 7974 meeting inclusion criteria (eFigure 1 in the Supplement). Data for 1 or more baseline covariates were missing for 789 participants (9.9%) (Table 1). In our study sample, 4023 participants (50.5%) were male, 3951 were female (49.5%), 2992 (37.5%) identified as Black, and 4982 (62.5%) identified as non-Black (median age at study entry, 18 [IQR, 17-20] years). Black and non-Black participants were similar in age distribution (eg, 21-22 years, 638 [21.3%] vs 1126 [22.6%]), sex (male, 1520 [50.8%] vs 2503 [50.2%]), and baseline disability (207 [6.9%] vs 260 [5.2%]). At baseline, non-Black participants were less likely to have a low income (median percentage of federal poverty level, 291% [IQR, 197%-414%] vs 142% [IQR, 85%-223%]) or receipt of public assistance (617 [12.4%] vs 908 [30.3%]), less likely to have not completed high school (697 [14.0%] vs 672 [22.5%]), and less likely to have at least 1 parent who did not complete high school (2206 [44.3%] vs 2245 [75.0%]). Black participants had a lower prevalence of past-year illicit drug use (1146 [41.0%] vs 2404 [51.3%]). At baseline, 219 participants (2.7%) had experienced at least 1 prior incarceration, which was less common among non-Black compared with Black participants (100 [2.0%] vs 119 [4.0%], respectively).

**Table 1.** Baseline Characteristics Overall and Stratified by Race^a^

Characteristic	Participant cohort		
	Full (N = 7974 [100%])	Black (n = 2992 [37.5%])	Non-Black (n = 4982 [62.5%])
Age in 1979, y			
15-16	1936 (24.3)	738 (24.7)	1198 (24.0)
17-18	2238 (28.1)	858 (28.7)	1380 (27.7)
19-20	2036 (25.5)	758 (25.3)	1278 (25.7)
21-22	1764 (22.1)	638 (21.3)	1126 (22.6)
Sex			
Female	3951 (49.5)	1472 (49.2)	2479 (49.7)
Male	4023 (50.5)	1520 (50.8)	2503 (50.2)
Disability^b^	467 (5.9)	207 (6.9)	260 (5.2)
Family income			
Median (IQR), % FPL^c^	230 (137-358)	142 (85-223)	291 (197-414)
Missing^b^	359 (4.5)	143 (4.8)	216 (4.3)
Receipt of public assistance	1525 (19.1)	908 (30.3)	617 (12.4)
Lack of HS completion by 22 y of age	1369 (17.2)	672 (22.5)	697 (14.0)
≥1 Parent did not complete HS^d^	4451 (55.8)	2245 (75.0)	2206 (44.3)
Illicit drug use^e^^,^^f^			
Yes	3550 (47.5)	1146 (41.0)	2404 (51.3)
No	3924 (52.5)	1646 (59.0)	2278 (48.7)
Missing^b^	500 (6.2)	200 (6.7)	300 (6.0)
Illegal activity^e^^,^^f^			
Yes	5111 (68.2)	1992 (71.1)	3119 (66.4)
No	2386 (31.8)	809 (28.9)	1577 (33.6)
Missing^b^	477 (6.0)	191 (6.4)	286 (5.7)
Baseline prior incarceration	219 (2.7)	119 (4.0)	100 (2.0)

Abbreviations: FPL, federal poverty level; HS, high school or equivalent.

^a^ Values in the table reflect the actual (unweighted) number of study participants. Unless otherwise indicated, data are expressed as number (%) of patients. Percentages are rounded and may not total 100%.

^b^ Baseline disability data were missing for 3 participants (2 Black and 1 non-Black). As a result of missingness for disability, income, illicit drug use, and illegal activity, a total of 789 participants had missing data for 1 or more baseline covariates.

^c^ Mean total annual family income from all available baseline surveys as a percentage of the year-specific FPL (mean during baseline period).

^d^ Includes participants with data missing for at least 1 parent.

^e^ Any past-year engagement in the activity, as self-reported on the 1980 survey.

^f^ Percentages for yes and no calculated without missing.

### Exposure to Incarceration

**Figure 1.**
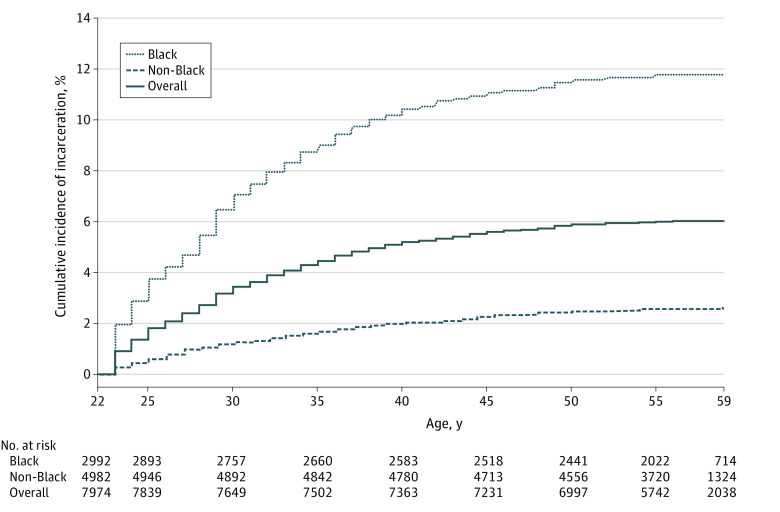
Cumulative Exposure to Incarceration (Accounting for Competing Risk of Death), Overall and Stratified by Race Cumulative incidence function estimates accounted for the competing risk of death to generate curves depicting the unadjusted cumulative incidence of exposure to follow-up incarceration over time. These curves depict the cumulative percentage of participants who had been exposed to at least 1 incarceration during follow-up. Regardless of any prior incarceration at baseline, all participants were considered unexposed to follow-up incarceration at baseline.

During a median follow-up time of 35 years (IQR, 33-37 years), 478 of 7974 participants (6.0%) were incarcerated at least once. Unadjusted cumulative incidence of incarceration was significantly higher among Black compared with non-Black participants throughout follow-up (χ^2^_1_ = 280.53; *P* < .001), and by 50 years of age, race-specific cumulative incidences of incarceration were 11.5% (95% CI, 10.4%-12.7%) and 2.5% (95% CI, 2.1%-2.9%), respectively (Figure 1). In the proportional subdistribution hazards model that accounted for competing risk of death, we found that the adjusted rate of incarceration exposure was higher among Black participants (adjusted HR [aHR], 3.47; 95% CI, 2.74-4.39) and among those who had prior incarceration at baseline (aHR, 5.88; 95% CI, 4.53-7.63), past-year illicit drug use (aHR, 1.55; 95% CI, 1.24-1.93), and past-year illegal activity (aHR 1.47; 95% CI, 1.10-1.96) (Table 2).

**Table 2.** Adjusted Hazards From Proportional Subdistribution Model for Time to Incarceration for the Full Cohort, Accounting for Competing Risk of Death (N = 7974)

Variable	aHR (95% CI)^a^
Baseline prior incarceration^b^	5.88 (4.53-7.63)
Race	
Non-Black	1 [Reference]
Black	3.47 (2.74-4.39)
Sex	
Female	1 [Reference]
Male	7.58 (5.58-10.30)
Disability^b^	1.37 (0.99-1.89)
Family income, % FPL^c^	0.82 (0.75-0.90)
Receipt of public assistance^b^	1.08 (0.85-1.38)
Lack of HS completion by 22 y of age	1.90 (1.53-2.36)
≥1 Parent did not complete HS^d^	1.39 (1.08-1.78)
Illicit drug use^e^	1.55 (1.24-1.93)
Illegal activity^e^	1.47 (1.10-1.96)

Abbreviations: aHR, adjusted hazard ratio; FPL, federal poverty level; HS, high school or equivalent.

^a^ Adjusted for all other variables in the table. Multiple imputation with 10 imputations was used to account for missing data among baseline covariates.

^b^ As reported during baseline period, that is, surveys completed before and during the year when a participant reached 22 years of age.

^c^ Mean baseline total family income is a continuous variable. We report the aHR associated with having a higher baseline income by an increment of 100% of the year-specific FPL.

^d^ Includes participants with data missing for at least 1 parent.

^e^ Includes any past-year engagement in the activity, as self-reported on the 1980 survey.

### Mortality

**Figure 2.**
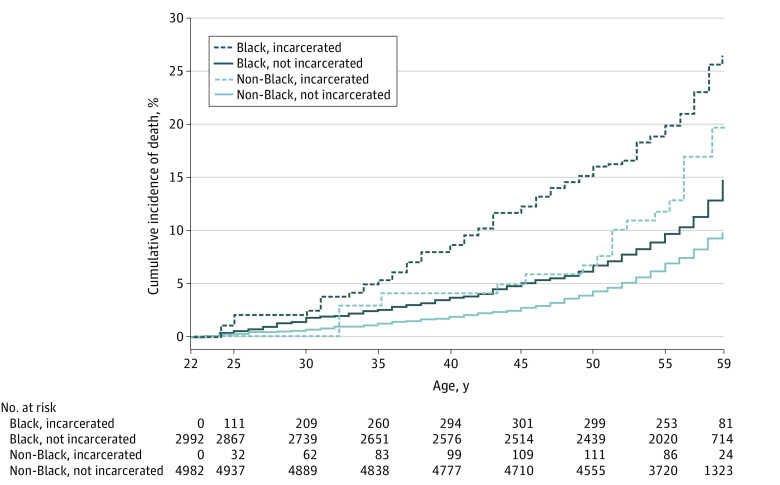
Extended Kaplan-Meier Cumulative Incidence of Death, Stratified by Race and Time-Varying Exposure to Incarceration The extended Kaplan-Meier estimator^26^ permits calculation of unadjusted cumulative incidence of death curves with a time-varying exposure to incarceration. Technically, extended Kaplan-Meier curves represent hypothetical cohorts whose exposure statuses remain constant throughout follow-up; because this method allows participants to transition between exposure groups during follow-up, we note that each curve cannot be technically interpreted as the percentage of a real cohort with an event over time.^27,28^

We identified 818 deaths (10.3% of sample) during follow-up. Based on data from the full cohort (eFigure 3 in the Supplement) and from each racial group (Figure 2), cumulative incidence curves using an extended Kaplan-Meier estimator demonstrated a consistently higher cumulative incidence of death among a hypothetical cohort of those with past incarceration exposure from the start compared with one whose members were never exposed. In the Cox proportional hazard regression models, time-varying incarceration was associated with an increased rate of death that was not statistically significant (aHR, 1.35; 95% CI, 0.97-1.88) (Table 3). Prior incarceration was not associated with mortality (aHR, 1.02; 95% CI, 0.65-1.60). Compared with non-Black participants, Black participants had significantly higher mortality rates (aHR, 1.28; 95% CI, 1.08-1.52; E-values, 1.88 for main estimate and 1.37 for lower bound of the 95% CI). In the analysis stratified by race, incarceration was the factor with the strongest association with mortality among Black participants (aHR, 1.65; 95% CI, 1.18-2.31; E-values, 2.68 for main estimate and 1.63 for the lower bound of the 95% CI), but this association was not significant among non-Black participants (aHR, 1.17; 95% CI, 0.68-2.03) (Table 3).

**Table 3.** Adjusted Hazards From the Multivariable Cox Regression Models of Death, Overall and Stratified by Race

Variables	Cohort, aHR (95% CI)^a^		
	Full (N = 7974)	Black (n = 2992)	Non-Black (n = 4982)
Incarceration during follow-up	1.35 (0.97-1.88)^b^	1.65 (1.18-2.31)^c^	1.17 (0.68-2.03)
Baseline prior incarceration^d^	1.02 (0.65-1.60)	0.80 (0.46-1.40)	1.12 (0.63-2.02)
Race			
Non-Black	1 [Reference]	NA	NA
Black	1.28 (1.08-1.52)^e^	NA	NA
Gender			
Female	1 [Reference]	1 [Reference]	1 [Reference]
Male	1.44 (1.21-1.70)	1.70 (1.31-2.21)	1.36 (1.11-1.66)
Disability^d^	1.45 (1.15-1.82)	1.67 (1.21-2.32)	1.35 (0.98-1.85)
Family income, % FPL^f^	0.98 (0.93-1.03)	1.04 (0.95-1.14)	0.97 (0.92-1.03)
Receipt of public assistance^d^	1.26 (1.02-1.57)	1.36 (1.09-1.70)	1.24 (0.92-1.68)
Lack of HS completion by 22 y of age	1.60 (1.33-1.92)	1.43 (1.11-1.83)	1.66 (1.29-2.13)
≥1 Parent did not complete HS^g^	1.22 (1.02-1.46)	1.49 (1.13-1.97)	1.18 (0.96-1.45)
Illicit drug use^h^	1.02 (0.87-1.19)	0.89 (0.71-1.12)	1.05 (0.87-1.27)
Illegal activity^h^	1.26 (1.02-1.56)	1.03 (0.83-1.27)	1.35 (1.02-1.77)

Abbreviations: aHR, adjusted hazard ratio; FPL, federal poverty level; HS, high school or equivalent; NA, not applicable.

^a^ Adjusted for all other variables in the table as well as for complex survey sampling design and sample weights. Multiple imputation with 10 imputations was used to account for missing data among baseline covariates.

^b^ For the association between incarceration and mortality in the full cohort analysis, the E-value was 2.04 for the main estimate (and because the 95% CI included the null, the E-value for the lower bound of the 95% CI is 1.00).

^c^ For the association between incarceration and mortality in the Black subgroup analysis, the E-values were 2.68 for the main estimate and 1.63 for the lower bound of the 95% CI.

^d^ As reported during baseline period, that is, surveys completed before and during the year when a participant reached 22 years of age.

^e^ For the association between Black (vs non-Black) race and mortality in the full cohort analysis, the E-values were 1.88 for the main estimate and 1.37 for the lower bound of the 95% CI.

^f^ Mean baseline total family income is a continuous variable. We report the aHR associated with having a higher baseline income by an increment of 100% of the year-specific FPL.

^g^ Includes participants with data missing for at least 1 parent.

^h^ Includes any past-year engagement in the activity, as self-reported on the 1980 survey.

Sensitivity analyses using complete case analysis (eTable 3 in the Supplement) and all non-Hispanic NLSY79 subsamples (eTable 4 in the Supplement) both had very similar findings to our main analysis. Sensitivity analysis with a single, unified time-varying incarceration exposure variable yielded incarceration-mortality associations that were stronger than those in our main analysis, including a statistically significant result in the full cohort model (aHR, 1.45; 95% CI, 1.12-1.88) (eTable 5 in the Supplement).

## Discussion

In a nationally representative, longitudinal cohort of 7974 non-Hispanic individuals who were followed up for nearly 4 decades, we found that experiencing an incarceration in adulthood was associated with lower life expectancy for Black but not for non-Black participants. Our study confirmed known racial disparities in rates of incarceration and life expectancy. Collectively, these data suggest that incarceration may be a key mediator of differential life expectancy between Black and non-Black populations in the US—a mediator that is a modifiable target for policy interventions.

Our study adds to the limited existing evidence regarding the association of incarceration with life expectancy and with racial health disparities. A prior study by Massoglia and colleagues^30^ that also used the NLSY79 data followed up participants to 47 years of age and found an association between incarceration and premature mortality only among women. Our study extends this previous one by including an additional decade of follow-up time (and nearly twice as many deaths) and by using survival analysis methods. In a retrospective cohort of all individuals released on parole from New York state prisons between 1989 and 1993, 1 study^31^ demonstrated that each additional year of prison time was associated with a nearly 2-year decline in postrelease life expectancy. However, most (>90%) of the sample was followed up for less than 4 years after release. Beyond the scarcity of relevant cohort studies, multiple recent studies using longitudinal ecological designs found associations between local increases in incarceration rates and increases in mortality among the whole population at the county level^12,32,33^ and among the bottom income quartile at the state level.^34^

Because very few prior studies have examined the contribution of incarceration to any kind of racial health disparities,^14^ our study provides the most direct evidence to date, to our knowledge, on the association between mass incarceration and racial disparities in life expectancy. Other studies using the NLSY79 data found that prior incarceration contributed marginally to racial disparities in disability^35^ and contributed substantially to disparities in general health functioning at 40 years of age.^36^ Furthermore, the results of our overall and race-stratified analysis indicate that the racial disparity of incarceration’s impact on health is driven by 2 mechanisms: (1) disparate rates of exposure to incarceration and (2) a disparate magnitude of effect on individuals who have been incarcerated. In addition, the fact that a higher risk of mortality remained for Black participants, even after we adjusted for socioeconomic and behavioral risk factors, suggests that our models do not adequately account for all the mechanisms by which structural racism creates health disparities. These could include tobacco and alcohol use, family wealth, broader community-level factors such as residential segregation, and any effects that are a function of cumulative exposure over time (because we only measured covariates at baseline).

With growing evidence of the negative association of incarceration with health disparities, ambitious, wide-ranging policy changes are needed to address the complex, mutually reinforcing factors that (re)produce both structural racism generally and mass incarceration specifically.^37,38,39^ Although reforms to criminal-legal systems themselves could help to mitigate the disparate consequences, substantial progress may be elusive without simultaneous improvements in interrelated areas such as economic opportunity, residential segregation, and mental health care, among others.^4^ To the extent that the social determinants of crime are also determinants of poor health,^40^ public health proponents can highlight how decarceration and greater investment in communities need not come at the expense of public safety.^41,42^ For the millions of individuals already affected by carceral systems, multidisciplinary supports are needed to better meet their compounded health and social needs.^43,44^ Health care clinicians should be able to engage with patients about personal histories of incarceration and understand the effects they can have on their health.^45^

### Limitations

Our study has several limitations. Because the NLSY79 cohort consists only of individuals born from 1957 to 1964, our results might not be generalizable to other birth cohorts. The NLSY79 race and ethnicity variable conflates 2 different types of measures (interviewer-perceived and self-reported), which resulted in a small amount of misclassification, although likely 5% or less (eMethods 2 and eTables 1 and 2 in the Supplement). Data on social, economic, and behavioral covariates were all based on self-report, which may introduce certain biases. Missingness of baseline covariate data may have been informative. However, fewer than 10% of participants had missing data, and results from the complete case sensitivity analysis were similar to those of the primary multiple imputation approach. Because our data source only detects incarcerations that occur at the time of an annual or biannual survey round, many incarcerations, especially shorter ones, are likely to be missed, leading to misclassification of exposure status; there is no way to know whether this misclassification differs in a systematic way that would bias our results.

Because many social and economic factors may function as mediators as well as confounders, we measured covariates only at baseline to avoid attenuating an association between incarceration and mortality by controlling for potential mediators. Limiting our main exposure variable to incarcerations after 22 years of age potentially limits the generalizability of our main analysis (and possibly underestimates incarceration’s full effect, as suggested by our sensitivity analysis with a unified incarceration variable [eTable 5 in the Supplement]), but we made this decision to avoid the potential bias of measuring the exposure before baseline covariates. Moreover, by including earlier incarcerations as a separate baseline variable, we were still able to include all the incarcerations in our data. Our analyses do not account for some potential baseline confounders (such as tobacco use, alcohol use, or family wealth, which were not surveyed in the earliest years of the NLSY79) nor for the possibility of time-dependent confounders.

## Conclusions

The findings of this study suggest that in the US, incarceration may contribute to the lower life expectancy of Black US residents. After 4 decades of mass incarceration, contact with the criminal-legal system is prevalent and unevenly distributed, and data are increasingly clear that it has a negative association with individual and population health. Efforts toward health equity and racial justice must consider the role of the criminal-legal system as a social determinant of health and a manifestation of structural racism.

## Notes

Supplement. **eMethods 1.** Notes on Inclusion/Exclusion of NLSY79 Subsamples **eMethods 2**. Choice of Race and Ethnicity Classification **eFigure 1**. Flowchart Depicting the Derivation of the Study Sample, With the Number of Individuals Meeting Each Exclusion Criterion **eFigure 2**. Timeline Depiction of Study Design **eFigure 3**. Extended Kaplan-Meier Cumulative Incidence Curve of Death, Stratified by Time-Varying Exposure to Follow-up Incarceration (Full Cohort, not Stratified by Race) **eTable 1.** Cross-tabulation of Self-reported Racial and Ethnic “Origin or Descent” (1979 Survey) With NLSY79’s Main Race and Ethnicity Categories **eTable 2.** Cross-tabulation of Self-reported Race and Ethnicity (2002 Survey) With NLSY79’s Main Race and Ethnicity Categories **eTable 3.** Complete Case Analysis: Adjusted Hazard Ratios (aHRs) From Multivariable Cox Models of Mortality Excluding Respondents With Any Missing Baseline Covariate Data **eTable 4.** Cohort Selection Sensitivity Analysis: Adjusted Hazard Ratios (aHRs) From Multivariable Cox Models of Mortality Using Data From All Non-Hispanic NLSY79 Subsamples **eTable 5.** Incarceration Variable Sensitivity Analysis: Adjusted Hazard Ratios (aHRs) From Multivariable Cox Models of Mortality Using a Single Time-Varying Incarceration Variable (Rather Than 2 Separate Variables) **eReferences**.
