# Supplementary material for: Association of Incarceration With Mortality by Race From a National Longitudinal Cohort Study
Source: JAMA Netw Open. 2021 Dec 23;4(12):e2133083. doi: 10.1001/jamanetworkopen.2021.33083 (PMC8703242; doi:10.1001/jamanetworkopen.2021.33083)
Supplement: Supplement. — eMethods 1. Notes on Inclusion/Exclusion of NLSY79 Subsamples eMethods 2. Choice of Race and Ethnicity Classification eFigure 1. Flowchart Depicting the Derivation of the Study Sample, With the Number of Individuals Meeting Each Exclusion Criterion eFigure 2. Timeline Depiction of Study Design eFigure 3. Extended Kaplan-Meier Cumulative Incidence Curve of Death, Stratified by Time-Varying Exposure to Follow-up Incarceration (Full Cohort, not Stratified by Race) eTable 1. Cross-tabulation of Self-reported Racial and Ethnic “Origin or Descent” (1979 Survey) With NLSY79’s Main Race and Ethnicity Categories eTable 2. Cross-tabulation of Self-reported Race and Ethnicity (2002 Survey) With NLSY79’s Main Race and Ethnicity Categories eTable 3. Complete Case Analysis: Adjusted Hazard Ratios (aHRs) From Multivariable Cox Models of Mortality Excluding Respondents With Any Missing Baseline Covariate Data eTable 4. Cohort Selection Sensitivity Analysis: Adjusted Hazard Ratios (aHRs) From Multivariable Cox Models of Mortality Using Data From All Non-Hispanic NLSY79 Subsamples eTable 5. Incarceration Variable Sensitivity Analysis: Adjusted Hazard Ratios (aHRs) From Multivariable Cox Models of Mortality Using a Single Time-Varying Incarceration Variable (Rather Than 2 Separate Variables) eReferences. [file jamanetwopen-e2133083-s001.pdf]

Supplementary Online Content

Bovell-Ammon BJ, Xuan Z, Paasche-Orlow MK, LaRochelle MR. Association of incarceration with mortality by race from a national longitudinal cohort study. *JAMA Netw Open*. 2021;4(12):e2133083. doi:10.1001/jamanetworkopen.2021.33083

eMethods 1. Notes on Inclusion/Exclusion of NLSY79 Subsamples

eMethods 2. Choice of Race and Ethnicity Classification

eFigure 1. Flowchart Depicting the Derivation of the Study Sample, With the Number of Individuals Meeting Each Exclusion Criterion

eFigure 2. Timeline Depiction of Study Design

eFigure 3. Extended Kaplan-Meier Cumulative Incidence Curve of Death, Stratified by Time-Varying Exposure to Follow-up Incarceration (Full Cohort, not Stratified by Race)

eTable 1. Cross-tabulation of Self-reported Racial and Ethnic “Origin or Descent” (1979 Survey) With NLSY79’s Main Race and Ethnicity Categories

eTable 2. Cross-tabulation of Self-reported Race and Ethnicity (2002 Survey) With NLSY79’s Main Race and Ethnicity Categories

eTable 3. Complete Case Analysis: Adjusted Hazard Ratios (aHRs) From Multivariable Cox Models of Mortality Excluding Respondents With Any Missing Baseline Covariate Data

eTable 4. Cohort Selection Sensitivity Analysis: Adjusted Hazard Ratios (aHRs) From Multivariable Cox Models of Mortality Using Data From All Non-Hispanic NLSY79 Subsamples

eTable 5. Incarceration Variable Sensitivity Analysis: Adjusted Hazard Ratios (aHRs) From Multivariable Cox Models of Mortality Using a Single Time-Varying Incarceration Variable (Rather Than 2 Separate Variables)

eReferences.

This supplementary material has been provided by the authors to give readers additional information about their work.

©2021 Bovell-Ammon BJ et al. *JAMA Network Open*.

eMethods 1. Notes on Inclusion/Exclusion of NLSY79 Subsamples

NLSY79 was initially comprised of a total of 12,686 participants from three independent probability samples: a cross-sectional sample of civilian population (n=6,111); supplemental oversamples of Black (n=2,172), Hispanic (n=1,480), and economically disadvantaged non-Black, non-Hispanic (n=1,643) participants; and a sample of participants in the military (n=1,280). The supplementary military subsample was specially funded by the Department of Defense (DOD), while NLSY79 generally has been funded and overseen by the Bureau of Labor Statistics in the Department of Labor. The DOD funding ended after the 1984 survey, and the military subsample was largely dropped at that point. However, to optimize the representativeness of the overall cohort, NLSY79 retained 201 members of the military subsample for ongoing, indefinite follow-up, since they complemented the civilian cross-sectional sample. Because these 201 retained participants remained eligible for ongoing follow-up through 2018 (or until deceased), we included them in our study sample. After the 1990 survey, due to funding limitations, NLSY79 dropped all 1,643 members of supplemental subsample of economically disadvantaged non-Black non-Hispanic participants. With these two subsample changes, a total of 2,722 military and civilian participants were dropped from the NLSY79 cohort early on (in the first decade), leaving 9,964 participants who have continued to be eligible for indefinite follow-up (i.e. into the present, since NLSY79 is still ongoing). For more details, see the information on NLSY79's web site (links below) as well as the Rothstein et al (2019) paper cited in the manuscript.

Because two of the supplemental subsamples (the military sample and the economically disadvantaged non-Black, non-Hispanic oversample) were prematurely dropped from the cohort (after 1984 and after 1990, respectively), we excluded these dropped participants from our study Even though it would have been possible to include such participants in our study (and administratively censor them if still alive when they are dropped), we wanted to focus on the cohort participants who were eligible for follow-up throughout all four decades of our study period (1979-2018), since this long-term follow-up duration was the most unique feature of the NLSY79 cohort data and of our specific study on the association between incarceration and health over the life-course. Including these individuals who only had a maximum of 5-12 years of follow-up would effectively weight our models toward this brief, early portion of our study period. Moreover, these dropped subsamples were different enough from other subsamples (particularly the military subsample) that we favored outright exclusion from our study on these grounds as well.

NLSY79 Web Site links:
Sample Design & Screening Process: https://nlsinfo.org/content/cohorts/nlsy79/intro-to-the-sample/sample-design-screening-process
Retention & Reasons for Non-interview: https://nlsinfo.org/content/cohorts/nlsy79/intro-to-the-sample/retention-reasons-noninterview

eMethods 2. Choice of Race and Ethnicity Classification

While we would have preferred to derive race/ethnicity information solely from respondents' self-identification and to be able to differentiate non-Hispanic White from other non-Hispanic non-Black groups, the self-reported data on race and ethnicity available in NLSY79 were not formulated in this manner. NLSY79's main race/ethnicity variable relied partly on an interviewer's "observation" (i.e., perception) of the participant's race in addition to self-reported information about their "origin or descent." In the (non-Hispanic) non-Black group, those who might have identified with a non-White race (e.g. Asian, Native American, Native Hawaiian/Pacific Islander) could not be consistently identified and collectively were of small numbers (likely less than 5% of the non-Hispanic non-Black group; see **eTables 1 and 2**), so we included them in the non-Black group in this study. Despite these two limitations, we felt that the most reproducible and unambiguous approach was to use the race/ethnicity variable provided by NLSY79. Cross-referencing the NLSY79-derived race/ethnicity variable with the available (though incomplete) self-reported data on race/ethnicity (from both 1979 and 2002; see below) suggests that the categories we used are reasonable approximations of those who self-identified as non-Hispanic Black and as non-Hispanic White, respectively. The information presented in this appendix summarizes information available from NLSY79's website (https://www.nlsinfo.org/content/cohorts/nlsy79/topical-guide/household/race-ethnicity-immigration-data)[1] and also references a helpful article by Light and Nandi (2007).[2]

Derivation of NLSY79's main race/ethnicity variable

NLSY79's race/ethnicity variable has three categories: Hispanic (of any race), non-Hispanic Black, and non-Hispanic non-Black. These categories were assigned based on information gathered in the pre-survey Household Screener, which was conducted in 1978 to define the sampling frame from which the cohort would be recruited the following year. Interviewers administered the screener to either the "youth" (a person born from 1957 to 1964 that might become a member of the NLSY79 cohort) or to the householder (typically a parent). Four specific items on the screener were used to categorize

race/ethnicity. First, interviewers were instructed to code a respondent's race "by observation" (i.e. according to the interviewer's own perception) as either "white," "black" or "other." Second, they asked respondents to select their "origin or descent" from a list of 15 categories shown on a hand card. Third, they asked if the head of the household or their spouse spoke a language other than English at home when they were a child. Fourth, they noted the family's surname. NLSY79 assigned Hispanic ethnicity if the respondent chose one of the Hispanic categories as their origin or descent, if the head of their household (or their spouse) spoke Spanish at home as a child, or if their family surname was on the US Census list of Spanish surnames. For non-Hispanic respondents, Black race was assigned if the race coded by the interviewer was Black or if the respondent chose "Black, Negro, Afro-American" as their origin or descent regardless of the interviewer's race coding. The remaining individuals, those in the non-Hispanic non-Black group, were predominantly non-Hispanic White individuals, but the data available does not provide a complete and precise way to isolate these individuals.

Cross-referencing NLSY79's main variable with self-reported variables

To test the accuracy of the NLSY79 race/ethnicity variable, here we compare it to self-reported data from survey items administered in 1979 and 2002. With the same wording that was used in the 1978 Household Screener, respondents were asked in 1979 (the first actual survey round) about their "origin or descent," but this time it was much more likely that the respondent was surveyed directly rather than the head of the household. There were almost twice as many response options (29 instead of 15), and they could endorse multiple (up to 6) and if multiple they were also asked, "Which one of these do you feel closest to?" Below, we present the only or primary ("closest") origin/descent reported by respondents in 1979 cross-tabulated by NLSY79 race/ethnicity categories (**eTable 1**). We also sorted 29 response options for origin/descent into 8 broader categories and calculate subtotals for these categories (**in bold**). Unfortunately, a non-negligible number of individuals have responses that are difficult to interpret, namely those who chose "Other," "None," or simply "American" (the latter being an option many respondents volunteered so it was coded specifically). Concerning the Native American category in the 1979 data, NLSY79's web site warns that is likely overestimated due to respondents' misinterpretation of the term(s) used in the questionnaire: "Users should be aware that frequency counts for the coding category "Indian American, or Native American are unusually high. About 5 percent of respondents reported this racial/ethnic origin, compared to Census estimates of approximately 0.5 percent of the population. This may have resulted from some respondents' misinterpretation of the term 'Native American'" (link: https://www.nlsinfo.org/content/cohorts/nlsy79/topical-guide/household/race-ethnicity-immigration-data). Besides these problematic/ambiguous responses, however, the remaining respondents in the non-Hispanic non-Black category overwhelmingly selected one of the options that we categorize as European descent, and the remaining respondents in the non-Hispanic Black category selected "Black" as their origin/descent.

In 2002 respondents were asked about their self-identified race and ethnicity using the new scheme implemented by the US Office of Management and Budget for the 2000 Census, where respondents could choose all the racial groups that they identify with and were asked separately about Hispanic ethnicity. However, since this was only administered during that one survey round, the rate of non-interview (not successfully interviewed that year) is about 20%, which is a common rate of non-interview for any given round (even respondents who are effectively retained in the cohort have missed one or more survey rounds over time since 1979). Nevertheless among those in our sample who responded to the 2002 race and ethnicity questions, an overwhelming majority of those in our non-Hispanic non-Black category self-identified their race as non-Hispanic White, and an overwhelming majority of those in our non-Hispanic Black category self-identified as non-Hispanic Black.

©2021 Bovell-Ammon BJ et al. *JAMA Network Open*.

eFigure 1. Flowchart Depicting the Derivation of the Study Sample, With the Number of Individuals Meeting Each Exclusion Criterion

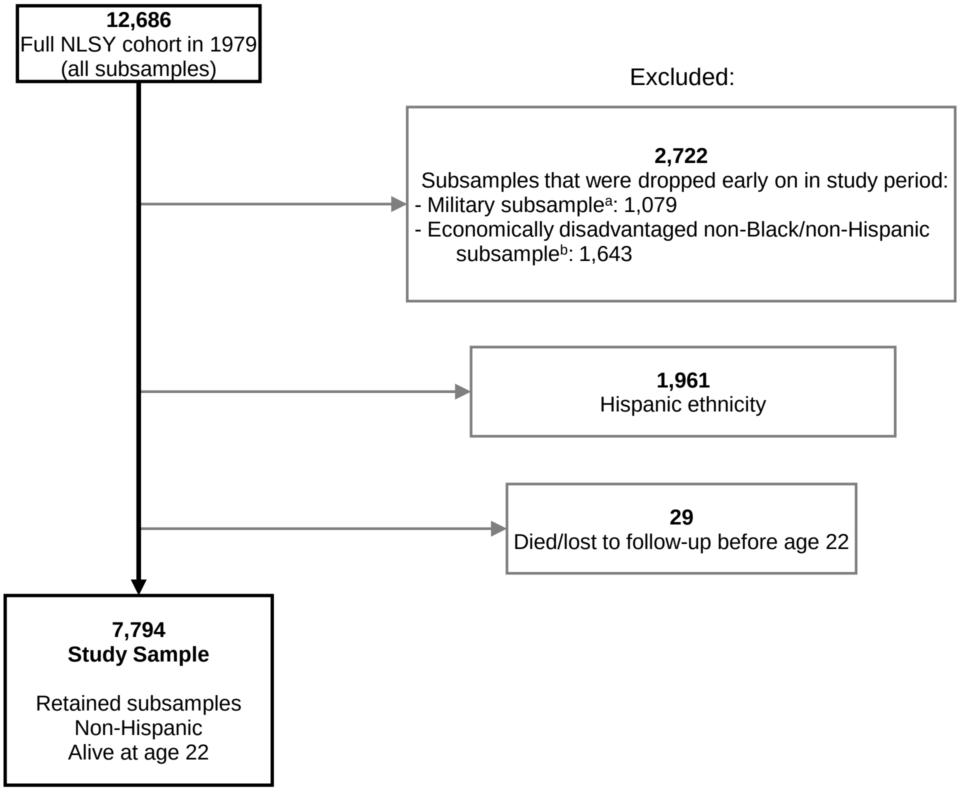

[a] The supplementary military subsample originally had 1,280 participants and was specially funded by the Department of Defense (DOD) (while NLSY79 generally has been funded and overseen by the Bureau of Labor Statistics in the Department of Labor. The DOD funding ended after the 1984 survey, and the military subsample was not followed after that. However, to maintain the overall representativeness of the cohort, NLSY79 retained 201 military subsample members for ongoing, indefinite follow-up since they would complement the civilian cross-sectional sample. Since these 201 retained participants remained eligible for ongoing follow-up through 2018 (or until deceased), we included them in our study sample.

[b] NLSY79 initially included supplemental samples (i.e. oversamples in addition to the national cross-sectional sample) of individuals from three disadvantaged groups. Specifically, the supplemental samples consisted of 2,172 Black individuals, 1,480 Hispanic individuals, and 1,643 economically disadvantaged non-Black/non-Hispanic individuals (predominantly non-Hispanic white). Due to subsequent funding limitations, NLSY79 dropped the economically disadvantaged non-Black/non-Hispanic individuals supplemental subsample after the 1990 survey. Due to its distinct composition and comparatively brief follow-up eligibility, we did not include these 1,643 participants in our study sample at all..

©2021 Bovell-Ammon BJ et al. *JAMA Network Open*.

eFigure 2. Timeline Depiction of Study Design

Figure is based on the format and terminology recommended by Schneewiss and colleagues[3] for depicting longitudinal study designs using databases and uses a free template which they made available online under a Creative Commons license at https://www.repeatinitiative.org/projects.html.

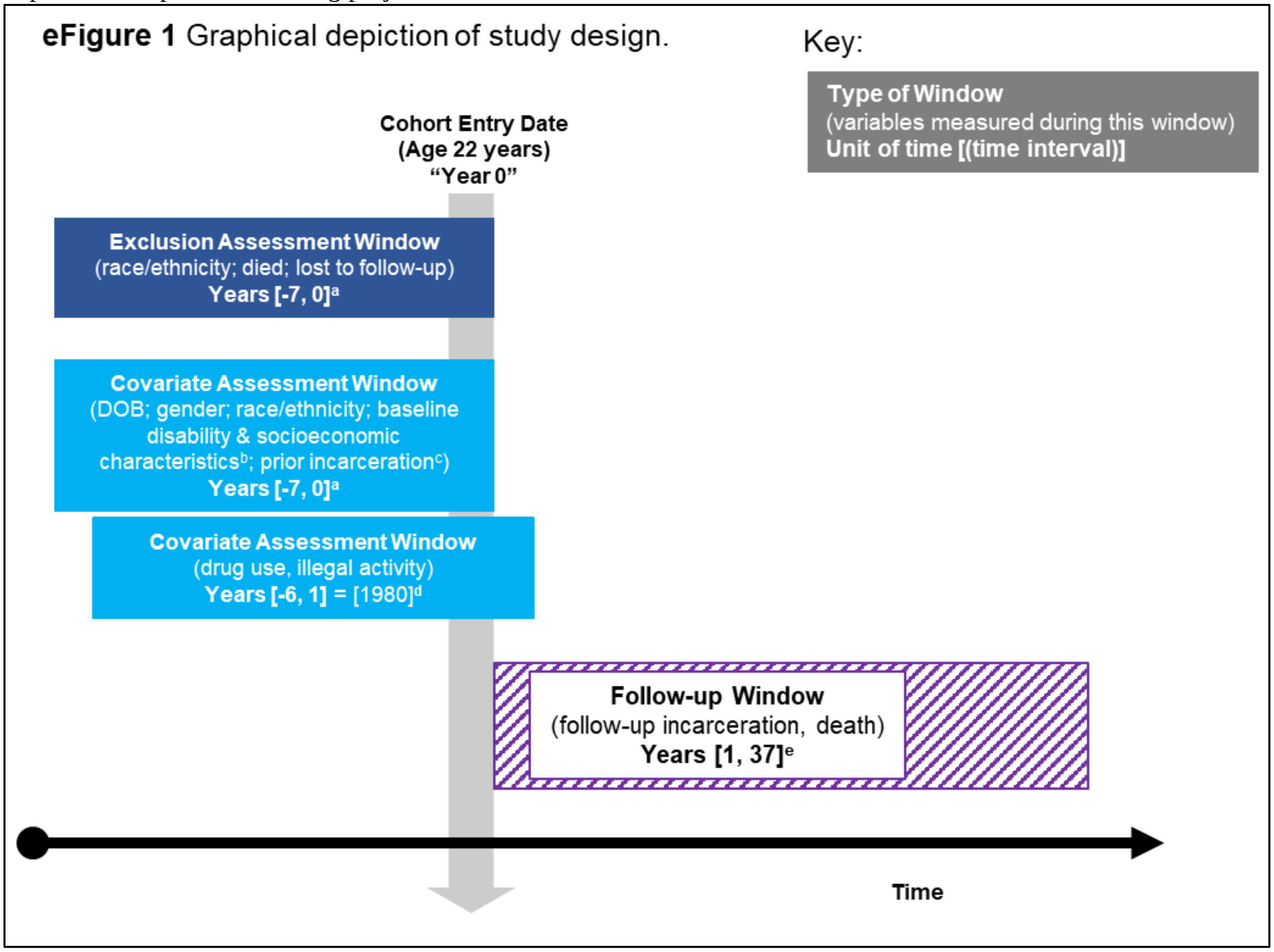

a. We used any and all available survey years prior to Cohort Entry Date (CED). Depending on their age in 1979 (the first NLSY survey year), respondents had a varying number of years of available surveys prior to CED, from 7 to 0. For example, if a respondent was already 22 in the 1979 survey then that was the CED and the only year available for exclusion assessment and baseline covariates. If a respondent was 15 in 1979 then they had 7 additional survey years prior to CED that we used for baseline data (and their CED corresponded with 1986 when they turned 22).
b. Baseline socioeconomic factors included family income, receipt of public assistance, whether respondent completed high school (or equivalent) by age 22, whether both of respondent's parents completed high school.
c. Any incarcerations that occurred at or prior to age 22 we considered to be "prior incarceration" at baseline.
d. Only the 1980 survey included data on prior drug use, violence, and illegal activity; so this survey year would correspond to Year -6 to Year 1, depending on respondent age.
e. We followed respondents until death or censoring (lost to follow up or end of source data range).

©2021 Bovell-Ammon BJ et al. *JAMA Network Open*.

eFigure 3. Extended Kaplan-Meier Cumulative Incidence Curve of Death, Stratified by Time-Varying Exposure to Follow-up Incarceration (Full Cohort, not Stratified by Race)

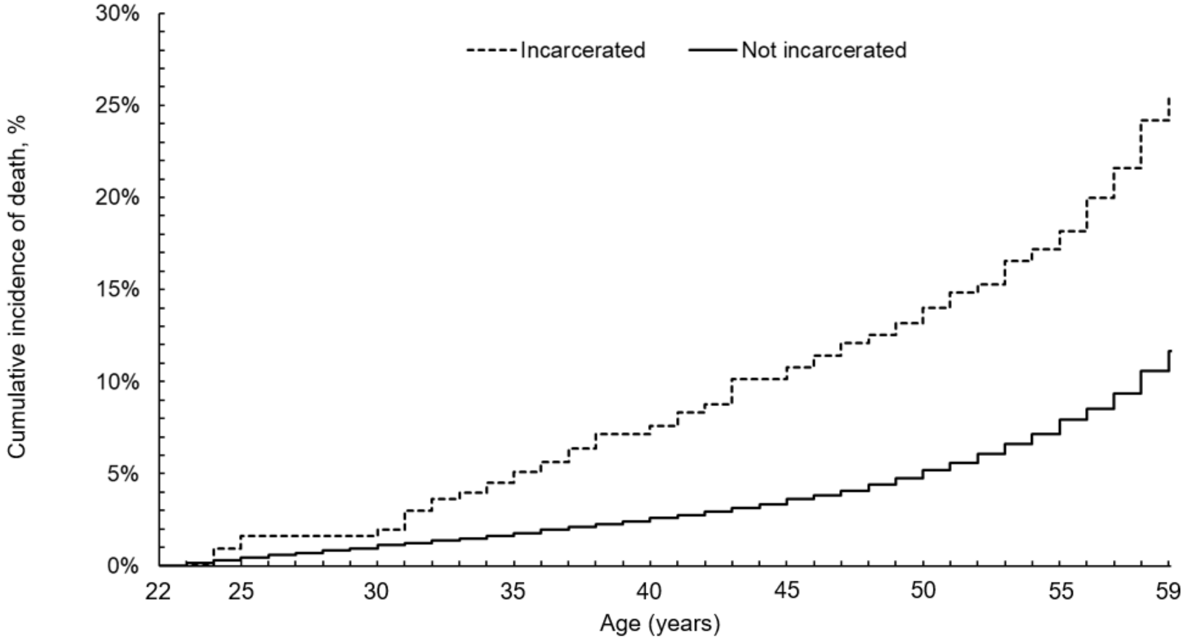

Number At Risk									
Overall, Incarcerated	0	143	271	343	393	410	410	339	105
Overall, Not incarcerated	7974	7804	7628	7489	7353	7224	6994	5740	2037

Legend: The extended Kaplan-Meier estimator[4] permits calculation of unadjusted cumulative incidence of death curves with a time-varying exposure to incarceration. Because this method allows participants to transition between exposure groups during follow-up, we note that each curve cannot be technically interpreted as the proportion of a distinct cohort with an event over time.[5,6] Nevertheless, the relative positions of the two curves indicate the relatively higher unadjusted mortality rate among those with prior follow-up incarceration.

©2021 Bovell-Ammon BJ et al. *JAMA Network Open*.

eTable 1. Cross-tabulation of Self-reported Racial and Ethnic "Origin or Descent" (1979 Survey) With NLSY79's Main Race and Ethnicity Categories

Here we compare this survey item's responses to the two main NLSY79 racial/ethnic categories included in our main analysis**,** non-Hispanic Black and non-Hispanic non-Black only. Note: Terms in all caps denote the original response options in the questionnaire, while the terms with asterisks (*) denote our own classifications that group together multiple questionnaire response options. The bold values in the table are the sums of all the individual responses contained in each group (for those that are grouped together).

	main NLSY79 race variable				Overall	
	non-Black (non-Hispanic)		Black (non-Hispanic)			
**Self-reported "origin or descent" on 1979 survey (only or primary option chosen)**	No.	column %	No.	column %	No.	column %
**BLACK**	**10**	**0.2%**	**2826**	**94.5%**	**2836**	**35.6%**
**European/White[a]**	**3353**	**67.3%**	**80**	**2.7%**	**3433**	**43.1%**
ENGLISH	976	19.6%	50	1.7%	1026	12.9%
FRENCH	176	3.5%	10	0.3%	186	2.3%
GERMAN	915	18.4%	5	0.2%	920	11.5%
GREEK	18	0.4%	0	0.0%	18	0.2%
IRISH	595	11.9%	3	0.1%	598	7.5%
ITALIAN	338	6.8%	6	0.2%	344	4.3%
POLISH	173	3.5%	3	0.1%	176	2.2%
PORTUGUESE	23	0.5%	3	0.1%	26	0.3%
RUSSIAN	33	0.7%	0	0.0%	33	0.4%
SCOTTISH	80	1.6%	0	0.0%	80	1.0%
WELSH	26	0.5%	0	0.0%	26	0.3%
**Hispanic[a]**	**23**	**0.5%**	**4**	**0.1%**	**27**	**0.3%**
CUBAN	0	0.0%	0	0.0%	0	0.0%
CHICANO	0	0.0%	0	0.0%	0	0.0%
MEXICAN	3	0.1%	0	0.0%	3	0.0%
MEXICAN-AMERICAN	9	0.2%	1	0.0%	10	0.1%
PUERTO RICAN	2	0.0%	1	0.0%	3	0.0%
OTHER HISPANIC	2	0.0%	0	0.0%	2	0.0%
OTHER SPANISH	7	0.1%	2	0.1%	9	0.1%
**Asian/NH/PI[a]**	**54**	**1.1%**	**10**	**0.3%**	**64**	**0.8%**
CHINESE	8	0.2%	4	0.1%	12	0.2%
FILIPINO	18	0.4%	4	0.1%	22	0.3%
ASIAN INDIAN	16	0.3%	1	0.0%	17	0.2%
JAPANESE	8	0.2%	0	0.0%	8	0.1%

©2021 Bovell-Ammon BJ et al. *JAMA Network Open*.

KOREAN	3	0.1%	1	0.0%	4	0.1%
VIETNAMESE	0	0.0%	0	0.0%	0	0.0%
HAWAIIAN, P.I.	1	0.0%	0	0.0%	1	0.0%
**INDIAN-AMERICAN OR NATIVE AMERICAN[b]**	**327**	**6.6%**	**17**	**0.6%**	**344**	**4.3%**
**"OTHER"**	**556**	**11.2%**	**17**	**0.6%**	**573**	**7.2%**
**"AMERICAN"**	**417**	**8.4%**	**10**	**0.3%**	**427**	**5.4%**
**None or Missing**	**241**	**4.8%**	**26**	**0.9%**	**267**	**3.3%**
"NONE"	78	1.6%	8	0.3%	86	1.1%
Missing	163	3.1%	18	0.6%	181	2.3%

Abbreviations: NH = Native Hawaiian; PI = Pacific Islander.
Note: terms in ALL CAPS = original response options in the questionnaire.
[a] Our own classifications which group together multiple original response options from the questionnaire. The bold values in the table are the sums of all the individual responses contained in each group (for those that are grouped together).
[b] NLSY79's web site warns that the number of respondents in the Native American category in 1979 data is likely overestimated due to respondents' misinterpretation of the term(s) used in the questionnaire: "Users should be aware that frequency counts for the coding category "Indian American, or Native American are unusually high. About 5 percent of respondents reported this racial/ethnic origin, compared to Census estimates of approximately 0.5 percent of the population. This may have resulted from some respondents' misinterpretation of the term 'Native American'" (link: https://www.nlsinfo.org/content/cohorts/nlsy79/topical-guide/household/race-ethnicity-immigration-data).

©2021 Bovell-Ammon BJ et al. *JAMA Network Open*.

eTable 2. Cross-tabulation of Self-reported Race and Ethnicity (2002 Survey) With NLSY79's Main Race and Ethnicity Categories

The 2002 survey used the updated race and ethnicity categories used by the Office of Management and Budget. Data from the 2002 race/ethnicity survey questions was missing for 1066 individuals in the non-Hispanic non-Black group (27.2% of that group in our sample) and for 627 individuals in the non-Hispanic Black group (26.5% of that group in our sample). Among those who have non-missing responses to the 2002 questions, we calculate percentages within their main race/ethnicity category (the categories we used in our analysis).

We include the number of missing (those in our sample who were not surveyed or did not answer this question in 2002) in each category.

	**NLSY79 race categories**				**Overall**	
	**non-Hispanic non-Black**		**non-Hispanic Black**			
**Self-reported race/ethnicity from 2002 survey**	No.	column % (of non-missing)	No.	column % (of non-missing)	No.	column % (of non-missing)
**Black**	10	0.3%	**2249**	**95.2%**	2259	36%
**White**	**3740**	**95.5%**	34	1.4%	3774	60%
**Hispanic (any race)**	54		18		72	
**Asian**	15	0.4%	4	0.2%	19	0%
**AIAN**	35	0.9%	3	0.1%	38	1%
**NHPI**	4	0.1%	1	0.0%	5	0%
**Other**	23	0.6%	11	0.5%	34	1%
**Multiple races**	34	0.9%	42	1.8%	76	1%
**Total** (non-missing)	3915	100%	2362	100%	6277	100%
**Number missing** (% of sample)	1066 (27.2% of total in our sample)		627 (26.5% of total in our sample)		1693 (27% of total in our sample)	

©2021 Bovell-Ammon BJ et al. *JAMA Network Open*.

eTable 3. Complete Case Analysis: Adjusted Hazard Ratios (aHRs) From Multivariable Cox Models of Mortality Excluding Respondents With Any Missing Baseline Covariate Data

Excluding the 789 participants with missing data for one or more baseline covariates produced very similar results to our main analysis which used multiple imputation.

	Full Cohort (n=7,185)			Stratified by Race					
				Black (n=2677)			non-Black (n=4508)		
Predictors	aHR[a]	(95% CI)		aHR[a]	(95% CI)		aHR[a]	(95% CI)	
**Incarceration (during follow-up)**	**1.35**	0.97	1.89	**1.76**	1.23	2.50	**1.15**	0.67	1.98
**Prior incarceration[b]**	**1.11**	0.70	1.79	**0.87**	0.47	1.59	**1.22**	0.67	2.22
**Race**									
non-Black	(ref)			-			-		
Black race	**1.21**	0.99	1.48	**-**			-		
**Gender**									
Female	(ref)			(ref)			(ref)		
Male	**1.43**	1.20	1.70	**1.57**	1.20	2.06	**1.38**	1.12	1.70
**Disability[b]**	**1.50**	1.17	1.93	**1.86**	1.36	2.55	**1.37**	0.96	1.94
**Family Income (%FPL)[c]**									
>300%	(ref.)			(ref.)			(ref.)		
130-300%	**1.12**	0.90	1.39	**1.41**	0.93	2.14	**1.08**	0.85	1.37
<130%	**1.23**	0.92	1.64	**1.31**	0.91	1.89	**1.31**	0.88	1.94
**Receipt of Public Assistance[b]**	**1.22**	0.96	1.54	**1.26**	0.98	1.61	**1.19**	0.86	1.66
**Lack of HS completion by 22**	**1.54**	1.24	1.92	**1.32**	1.01	1.74	**1.62**	1.22	2.15
**Parents did not both complete HS[d]**	**1.23**	1.00	1.50	**1.39**	1.03	1.89	**1.20**	0.95	1.51
**Illicit drug use[e]**	**0.95**	0.81	1.12	**1.17**	0.92	1.49	**0.90**	0.74	1.11
**Illegal activity[e]**	**0.75**	0.60	0.94	**0.91**	0.74	1.13	**0.71**	0.54	0.94

Abbreviations: CI = 95% confidence interval; %FPL = percent of year-specific federal poverty level; ref. = Reference group; HS = high school or equivalent.

[a] Adjusted for all other variables in the table as well as for complex survey sampling design and sample weights. We also used multiple imputation (with 10 imputations) to account for missing data among baseline covariates.

[b] As reported during baseline period, i.e. surveys completed prior to and during the year when a participant reached age 22

[c] Mean total annual family income from all available baseline surveys

[d] If at least one parent had not completed high school or data was missing for at least one parent

[e] Any past-year engagement in the activity, as self-reported on the 1980 survey

©2021 Bovell-Ammon BJ et al. *JAMA Network Open*.

eTable 4. Cohort Selection Sensitivity Analysis: Adjusted Hazard Ratios (aHRs) From Multivariable Cox Models of Mortality Using Data From All Non-Hispanic NLSY79 Subsamples

As stated in eAppendix 1, there were two NLSY79 subsamples (the military sample and the economically disadvantaged non-Black non-Hispanic supplemental oversample) that were dropped (after the 1984 and 1990 surveys, respectively) due to changes in funding for the study, i.e. purely administrative reasons. Since these subsamples were only eligible for very brief maximum duration of follow-up (5 and 11 years, respectively), we excluded them entirely from our study sample, since their person-years would effectively weight our long-term mortality models more toward the early years when death rates are very low. To assess the impact of this decision to exclude these two subsamples, we performed a sensitivity analysis with the full cohort, i.e. all non-Hispanic subsamples/participants (n=10,649, compared to 7,974 in our main analysis). These results are essentially identical to those of our main analysis.

	**Full Cohort** *n=10,649*			**Stratified by Race**					
				**Black** *n=3,165*			**non-Black** *n=7,484*		
Variables	**aHR**[a]	95% CI		**aHR**[a]	95% CI		**aHR**[a]	95% CI	
**Incarceration (during follow-up)**	**1.35**	0.97	1.87	**1.64**	1.17	2.31	**1.17**	0.68	2.02
**Baseline prior incarceration**[b]	**1.02**	0.65	1.60	**0.80**	0.46	1.40	**1.12**	0.63	2.00
**Race**									
non-Black	(ref.)			-			-		
Black	**1.29**	1.09	1.52	-			-		
**Gender**									
Female	(ref.)			(ref.)			(ref.)		
Male	**1.44**	1.21	1.70	**1.71**	1.31	2.23	**1.36**	1.11	1.66
**Disability**[b]	**1.44**	1.15	1.81	**1.67**	1.21	2.31	**1.34**	0.97	1.83
**Family Income (%FPL)**[c]	**0.98**	0.93	1.03	**1.03**	0.94	1.12	**0.97**	0.92	1.03
**Receipt of Public Assistance**[b]	**1.26**	1.02	1.57	**1.36**	1.09	1.70	**1.24**	0.92	1.68
**Lack of HS completion by 22**	**1.58**	1.32	1.90	**1.42**	1.11	1.82	**1.64**	1.28	2.10
**Parents did not both complete HS**[d]	**1.23**	1.03	1.47	**1.49**	1.13	1.95	**1.19**	0.97	1.46
**Illicit drug use**[e]	**1.03**	0.88	1.21	**0.91**	0.71	1.16	**1.06**	0.88	1.29
**Illegal activity**[e]	**1.27**	1.04	1.56	**1.01**	0.81	1.25	**1.37**	1.06	1.77

Abbreviations: 95% CI = 95% confidence interval; ref. = Reference group; %FPL = percent of year-specific federal poverty level; HS = high school or equivalent.

[a] Adjusted for all other variables in the table as well as for complex survey sampling design and sample weights. We also used multiple imputation with 10 imputations to account for missing data among baseline covariates.

[b] As reported during baseline period, i.e. surveys completed prior to and during the year when a participant reached age 22

[c] Mean baseline total family income is a continuous variable. We report the adjusted hazard ratio associated with having a higher baseline income by an increment of 100% FPL.

[d] If at least one parent had not completed high school or data was missing for at least one parent

[e] Any past-year engagement in the activity, as self-reported on the 1980 survey

©2021 Bovell-Ammon BJ et al. *JAMA Network Open*.

eTable 5. Incarceration Variable Sensitivity Analysis: Adjusted Hazard Ratios (aHRs) From Multivariable Cox Models of Mortality Using a Single Time-Varying Incarceration Variable (Rather Than 2 Separate Variables)

We considered an alternative design of the exposure variable using a single time-varying incarceration exposure variable that captured incarcerations at all ages (i.e. individuals who had already been incarcerated at or before age 22 were classified as exposed from the start of the follow-up period).

	Full Cohort			Stratified by Race					
				Black			non-Black		
	n=	*7974*		*n=*	*2992*		*n=*	*4982*	
Variables	**aHR**[a]	(95% CI)		**aHR**[a]	(95% CI)		**aHR**[a]	(95% CI)	
**Incarceration**	**1.45**	1.12	1.88	**1.61**	1.19	2.17	**1.37**	0.92	2.05
**Race**									
non-Black	(ref.)			-			-		
Black	**1.28**	1.08	1.52	-			-		
**Gender**									
Female	(ref.)			(ref.)			(ref.)		
Male	**1.41**	1.19	1.67	**1.67**	1.28	2.17	**1.34**	1.10	1.64
**Disability**[b]	**1.46**	1.16	1.83	**1.68**	1.21	2.33	**1.36**	0.99	1.86
**Family Income (%FPL)**[c]	**0.98**	0.93	1.03	**1.04**	0.95	1.14	**0.97**	0.92	1.03
**Receipt of Public Assistance**[b]	**1.24**	1.01	1.54	**1.35**	1.08	1.69	**1.23**	0.91	1.66
**Lack of high school completion (by age 22)**	**1.56**	1.30	1.89	**1.39**	1.08	1.79	**1.63**	1.26	2.10
**Parents did not both complete high school**[d]	**1.22**	1.02	1.45	**1.50**	1.13	1.98	**1.17**	0.95	1.44
**Illicit drug use**[e]	**1.01**	0.86	1.18	**0.88**	0.70	1.11	**1.04**	0.86	1.26
**Illegal activity**[e]	**1.26**	1.01	1.56	**1.01**	0.81	1.26	**1.34**	1.02	1.76

Abbreviations: 95% CI = 95% confidence interval; ref. = Reference group; %FPL = percent of year-specific federal poverty level.

[a] Adjusted for all other variables in the table as well as for complex survey sampling design and sample weights. We also used multiple imputation (with 10 imputations) to account for missing data among baseline covariates.

[b] As reported during baseline period, i.e. surveys prior to and during the year when a participant reached age 22

[c] Mean baseline total family income is a continuous variable. We report the adjusted hazard ratio associated with having a higher baseline income by an increment of 100% FPL.

[d] If at least one parent had not completed high school or data was missing for at least one parent

[e] Any past-year engagement in the activity, as self-reported on the 1980 survey

©2021 Bovell-Ammon BJ et al. *JAMA Network Open*.

eReferences

1. U.S. Bureau of Labor Statistics. Race, Ethnicity & Immigration Data (NLSY79). National Longitudinal Surveys. Accessed February 5, 2021. https://www.nlsinfo.org/content/cohorts/nlsy79/topical-guide/household/race-ethnicity-immigration-data
2. Light A, Nandi A. Identifying race and ethnicity in the 1979 National Longitudinal Survey of Youth. *Population Research and Policy Review*. 2007;26(2):125. doi:http://dx.doi.org.ezproxy.bu.edu/10.1007/s11113-007-9021-1
3. Schneeweiss S, Rassen JA, Brown JS, et al. Graphical Depiction of Longitudinal Study Designs in Health Care Databases. *Ann Intern Med*. 2019;170(6):398. doi:10.7326/M18-3079
4. Snapinn SM, Jiang Q, Iglewicz B. Illustrating the Impact of a Time-Varying Covariate with an Extended Kaplan-Meier Estimator. *The American Statistician*. 2005;59(4):301-307.
5. Sjölander A. A Cautionary Note on Extended Kaplan–Meier Curves for Time-varying Covariates. *Epidemiology*. 2020;31(4):517-522. doi:10.1097/EDE.0000000000001188
6. Beyersmann J, Gerds T, Schumacher M. Letter to the editor: comment on ‘Illustrating the impact of a time-varying covariate with an extended Kaplan-Meier estimator’ by Steven Snapinn, Qi Jiang, and Boris Iglewicz in the November 2005 issue of The American Statistician. *The American Statistician*. 2006;60(3):295-297. doi:10.1198/000313006X118700

©2021 Bovell-Ammon BJ et al. *JAMA Network Open*.
